# Influence of Deep Eutectic Solvent Composition on Micelle Properties: A Molecular Dynamics Study

**DOI:** 10.3390/molecules30030574

**Published:** 2025-01-27

**Authors:** Iuliia V. Voroshylova, Elisabete S. C. Ferreira, M. Natália D. S. Cordeiro

**Affiliations:** REQUIMTE LAQV, Department of Chemistry and Biochemistry, Faculty of Sciences, University of Porto, 4169-007 Porto, Portugal; elisabete.ferreira@fc.up.pt (E.S.C.F.); ncordeir@fc.up.pt (M.N.D.S.C.)

**Keywords:** Ethaline, Glyceline, Reline, surfactant, micelle, MD simulations, hydrogen bonding, sodium dodecyl sulfate, sulfobetaine, cetyltrimethylammonium bromide

## Abstract

This study investigates the structural and transport properties of SDS, CTAB, and SB3-12 micelles in three deep eutectic solvents (DESs), Ethaline, Glyceline, and Reline, using molecular dynamics (MD) simulations. The influence of solvent composition on micelle morphology, interactions, and dynamics was explored, revealing key differences driven by the DES environment. Structural analyses, including eccentricity and radius of gyration, demonstrated that micelle shape and compactness vary significantly depending on the solvent. In Ethaline and Reline, larger micelles showed significant deviations from spherical shapes, while micelles in Glyceline became more spherical and compact, particularly those formed by SB3-12. Radial distribution functions highlighted different levels of micelle–solvent interactions, with SDS showing strong interactions with HBD components and SB3-12 exhibiting prominent self-interaction. According to hydrogen bonding analysis, micelles slightly disrupt the DES hydrogen bond network, with SB3-12 establishing the most significant hydrogen bond connections. The transport property analysis revealed that larger micelles have lower diffusion coefficients, whereas smaller micelles enhance DESs’ component mobility. These findings advance the understanding of micelle behavior in DESs and also help in the optimization of DES–surfactant systems for applications such as electrodeposition, nanomaterial templating, and drug delivery. Future research will focus on surfactant interactions with surfaces to further improve these applications.

## 1. Introduction

Deep eutectic solvents (DESs) represent a promising class of sustainable solvents, combining a hydrogen bond acceptor (HBA), typically an organic salt, with a hydrogen bond donor (HBD), such as an alcohol or amide [1]. This combination creates a stable liquid phase at relatively low temperatures through a network of strong hydrogen bonds [2,3]. Common HBA–HBD combinations often have specific names; for example, choline chloride (ChCl) combined with ethylene glycol (Eg) in a 1:2 molar ratio forms the DES known as Ethaline [4]. Similarly, ChCl paired with urea (Ure) as the HBD produces Reline [5], while with glycerol (Gly), it forms Glyceline [6]. DESs share several properties with ionic liquids (ILs), such as high ionic strength, low volatility, and thermal stability [7,8,9,10,11], but offer environmental advantages, cost-effectiveness, and straightforward preparation [1,6,9,12,13]. By carefully selecting HBA and HBD components, properties like viscosity, polarity, and conductivity can be finely tuned [2,14], making DESs highly adaptable for applications in electrochemistry, catalysis, and bio-related fields, where they can stabilize biomolecules and enhance solubility [3,8,13,15,16,17,18,19,20,21,22,23,24,25].

Incorporating surfactants such as sodium dodecyl sulfate (SDS), cetyltrimethylammonium bromide (CTAB), and N-dodecyl-N,N-dimethyl-3-ammonio-1-propanesulfonate (SB3-12) into DESs has broadened the potential applications of these solvents, especially in areas requiring controlled self-assembly, such as nanomaterial templating and drug delivery [26,27,28,29]. Notably, surfactants in DESs can play a critical role in electrodeposition applications, where their aggregation properties influence deposit morphology and surface characteristics [29,30,31,32]. For instance, SDS enhances copper nanoparticle electrodeposition in Reline [30]. In Ethaline, CTAB produces a smooth, nearly globular morphology in electrodeposited Au layers, whereas SDS yields Au coatings with sharp-edged facets [31]. Lately, Häckl et al. modified the surface of graphite electrodes with CTAB and SDS surfactants in Ethaline and Glyceline [29]. These DES–surfactant systems may optimize electrodeposition by modulating surface interactions, facilitating the production of well-defined nanostructured materials, and widening electrochemical windows [29,32].

The unique self-assembly behavior of surfactants in DESs arises from their amphiphilic nature, which drives micelle formation in polar solvents once the critical micelle concentration (CMC) is reached. In these micelles, hydrophobic tails are encapsulated within a polar solvent shell, effectively reducing the system’s free energy [33,34,35]. The specific DES composition further influences solubility and micelle morphology; for example, while CTAB is insoluble in Reline and Ethaline, it forms stable micelles in Glyceline [36,37,38].

Micelle structure and stability in DESs can differ notably from those in water. In Reline, for instance, SDS forms cylindrical rather than spherical micelles as observed by Arnold et al. [39]. In Reline–Glyceline mixtures, both SDS and CTAB display shape transitions, forming elongated micelles at low glycerol content and globular aggregates as glycerol content increases, with intermediate shapes seen during this transition. This change in shape, attributed to headgroup solvation and counterion binding at the micelle surface, demonstrates how solvent composition can fine-tune self-assembly properties [29,36].

Hammond et al. suggest that differences in micelle shape can also be influenced by solvent penetration, which alters micelle characteristics [40]. In pure Glyceline, CTAB micelles maintain shapes and sizes similar to those in water [37], while SB3-12 forms globular micelles [28]. Additionally, the high ionic strength of DESs can weaken headgroup interactions within micelles, potentially leading to larger, more stable aggregates than those typically found in aqueous systems [37]. Meanwhile, in aqueous DESs, such as choline chloride with water (known as Aquiline), surfactant behavior supports environmentally friendly electrochemical processes [41].

Molecular dynamics (MD) simulations can offer critical insights into the behavior of these surfactant–DES systems on a molecular level. Numerous studies have applied MD to understand SDS, CTAB, and other surfactants’ aggregation, solubilization properties, and interactions in aqueous environments, laying a foundation for DES studies [42,43,44,45,46,47,48,49,50,51,52,53,54,55,56,57,58,59,60,61,62,63,64,65,66,67,68,69,70,71,72,73,74,75,76,77,78]. For example, studies such as [43,45,48,50,69] investigated SDS and CTAB micellization in water, while others explored specific interactions with additives or at interfaces, such as aromatic molecule solubilization [59], micelle aggregation [53], and phenol adsorption on solid surfaces [60]. These MD simulations provide a comparative framework for exploring surfactant organization and dynamics within DESs, which introduce additional factors such as DES-specific hydrogen bonding, counterion effects, and interaction with nanoparticles or surfaces [44,52,54,55,65,66,70,75,78].

This study addresses the behavior and characteristics of SDS, CTAB, and SB3-12 micelles in bulk DES environments, specifically in Ethaline, Glyceline, and Reline, using MD simulations. Understanding micelle behavior in bulk is a crucial step towards tailoring surface modifications with surfactants in DESs for advanced applications in electrodeposition. Furthermore, insights from this study can benefit broader applications, including sustainable nanomaterials templating, biocompatible formulations, and drug delivery systems, as DES-based self-assembly strategies continue to expand.

## 2. Computational Details

### 2.1. Simulation Setup

All molecular dynamics (MD) simulations were conducted using GROMACS 2020.4 [79]. For the DESs in this study, the best-performing force fields (FFs) were selected to ensure accuracy. Specifically, an OPLS-based FF previously refined by our group was applied to Ethaline [4], while the Glyceline and Reline systems utilized the FF developed by Doherty and Acevedo [80] without further modification. To maintain compatibility across DES systems, surfactant molecules were modeled using the OPLS-AA transferable force field [81]. These force fields were chosen for their reliable performance in replicating experimental physicochemical properties of pure fluids and surfactant systems [6,66,80,82,83].

**Figure 1:**
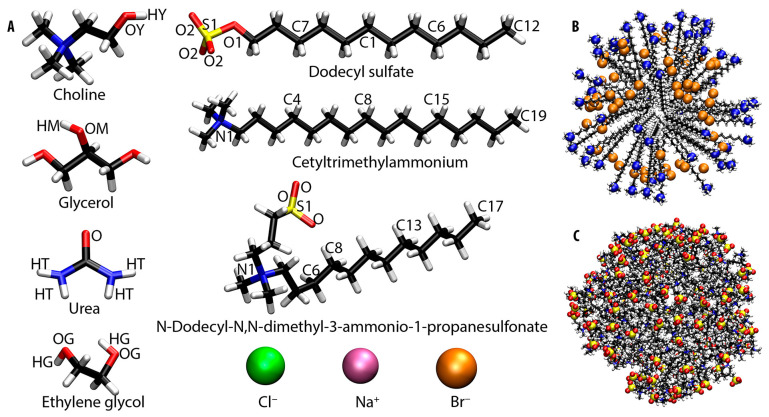
(**A**) Chemical structures of choline cation, glycerol, urea, ethylene glycol, dodecyl sulfate, cetyltrimethylammonium, N-dodecyl-N,N-dimethyl-3-ammonio-1-propanesulfonate, chloride anion, bromide anion, and sodium cation; (**B**) structure of initial CTAB m60 micelle configuration; (**C**) structure of SB3-12 m120 micelle after production run. Carbon, nitrogen, oxygen, hydrogen, sulfur, chloride, bromide, and sodium atoms are represented by black, blue, red, white, yellow, green, orange, and pink colors, respectively.

Charges for optimized surfactant configurations were derived using the restrained electrostatic potential (RESP) fit procedure in Gaussian 16 [84]. Figure 1A provides a schematic representation of all simulated species with indication of all relevant atom types.

To investigate the influence of surfactant concentration on micellar microstructure, systems with two micelle sizes were constructed. Spherical micelles containing either 60 (small, m60) or 120 (large, m120) surfactant molecules were built using Packmol [85], with hydrophilic heads facing outward and hydrophobic tails inward. The smaller micelles (with an aggregation number of 60) reflect typical values reported in experimental [27,41,86,87] and computational studies [48,50,54,55,64,69,77]. Each micelle was then positioned at the center of a simulation box containing 1024 choline chloride ion pairs and 2048 HBD molecules. Table 1 provides details on both the system composition and equilibrated box dimensions, while Figure 1B,C show snapshot examples of the initial and final configurations of micelles, respectively.

Energy minimization was first performed with the steepest-descent algorithm to ensure system relaxation, targeting a maximum force tolerance of 10 kJ mol^−1^ nm^−1^. Following this, two pre-equilibration steps were conducted in the canonical ensemble (100 ps at 100 K) and in the isothermal–isobaric ensemble (500 ps at 298.15 K). A subsequent annealing procedure in the NVT ensemble was carried out over 2 ns, involving heating to 350 K, maintaining that temperature for 1 ns, and cooling back to 298.15 K to overcome potential energy barriers. Integration times of 0.5 fs were used in all these steps, with no bond constraints applied. Final equilibration (10 ns in NpT) and production runs (50 ns in NVT) were conducted at 298.15 K and 1 bar (for equilibration), with coordinates recorded every 1000 steps. During these steps, a 1 fs time step was applied, and bonds with hydrogens were constrained using the LINCS algorithm [88]. For the initial pre-equilibration, temperature and pressure were controlled by the robust v-rescale thermostat [89] and Berendsen barostat [90], each with a 0.5 ps coupling constant. For the remainder of the simulation, temperature and pressure control were switched to the Nosé–Hoover thermostat [91,92] and Parrinello–Rahman barostat [93], with coupling constants set to 2.0 ps and 8.0 ps, respectively. The leapfrog algorithm [94] was used for integration, with long-range electrostatics handled via the particle-mesh Ewald method [95] and a 1.2 nm cut-off applied for neighbor lists and short-range interactions. Long-range van der Waals interactions were adjusted using a continuum model correction. Periodic boundary conditions were enforced in all three dimensions.

To explore surfactant solvation energetics in DES, we calculated solvation free energy, Δ*G*, using the Free Energy Perturbation (FEP) method [96,97,98,99]. Systems containing a single surfactant molecule were equilibrated, following the same protocol as for the micelle systems. For solvation free energy calculations, surfactant charges were set to zero in the topology file to avoid instability from charge attraction. Transformation between states (from gas phase to solvated surfactant) was governed by a coupling parameter, *λ*, ranging from 0.0 to 1.0 across 21 stages. The Langevin integrator was used to avoid excessive damping of the fluid dynamics. In the NpT equilibration, the Parrinello–Rahman barostat was used to maintain the pressure at 1 bar, while the thermostat was implicitly handled by the integrator.

Density, *d*; self-diffusion coefficients, *D*; radial distribution functions (RDFs), *g*(*r*); and hydrogen bonding interactions (Hbonds) were calculated using GROMACS in-built tools. Self-diffusion coefficients were validated by confirming diffusive regimes via the parameter *β*, as described elsewhere [3,4,82,100,101]. Where applicable, error estimations for simulated versus experimental values were determined as |*X*_exp_ − *X*_sim_|/*X*_exp_ × 100%, where *X*_exp_ is the experimentally obtained value, and *X*_sim_ is the computationally obtained value. Visual Molecular Dynamics (VMD) [102] package was employed for hydrogen bond analysis and visualization.

### 2.2. Validation

The first step in the simulation process is validating the FF models used in the study. Since the FF for Ethaline had already been developed and validated by our group [4], no further validation was required. Therefore, validation was only conducted for the FFs used for Glyceline and Reline. For this purpose, additional MD runs for pure Glyceline and Reline systems, each containing 1024 ion pairs of ChCl and 2048 molecules of the respective HBD (glycerol or urea), were conducted using the MD protocol described earlier. All FFs employed here are based on the OPLS FF, originally calibrated using thermodynamic properties [81]. To assess their suitability, we calculated density at 298.15 K and, though this study focuses on structural properties, we also evaluated self-diffusion coefficients. The calculated (sim) densities and self-diffusion coefficients are presented in Table 2, in comparison with their experimental values (exp) when possible.

As shown in Table 2, the simulated density values align well with experimental data, showing average errors of 0.5% for Glyceline and 6% for Reline. For self-diffusion coefficients, the results serve as a qualitative indicator of the trends, rather than precise values of transport properties, given the challenges in accurately capturing transport behavior in highly viscous liquids such as DESs, especially at room temperature. Reline, for instance, has a reported viscosity of 515 cP [80] and 1398 cP [105] at 298.15 K, making it difficult to achieve a true diffusive regime for such highly viscous liquid at room temperature. Consequently, values reported in Table 2 for Reline were obtained at 373.15 K, a temperature that allowed for a diffusive regime, as shown by *β* values in Figure S1 of the Supporting Information (SI). Although no experimental self-diffusion data for Reline are available at 373.15 K, the reported values at 328 K (the highest temperature at which self-diffusion coefficient values were reported) are 2.10·10^11^ m^2^ s^−1^ for Ch^+^ and 3.55·10^11^ m^2^ s^−1^ for urea [106]. Since diffusion increases with temperature, experimental values at 373.15 K are expected to be higher than our simulation results, which is consistent with the FF limitations noted by Doherty and Acevedo for transport properties at room temperature [80].

For Glyceline, which is substantially less viscous (259 cP at 298.15 K [106]), a diffusive regime at 298.15 K was achieved, as shown by the *β* parameter in Figure S1 of the SI, allowing us to report self-diffusion values at this temperature. The trends observed in both DESs remain consistent: as expected, self-diffusion coefficients for Ch^+^ are lower than those for the HBD [2,80,103,104,105,106]. Additionally, the water content in choline-chloride-based DESs, which are hygroscopic, significantly impacts experimental properties [107,108]. It should be noted that these simulations do not account for water content, so differences between experimental and simulated values are anticipated. Nevertheless, given the consistent trends in density and self-diffusion and the study’s focus on microscopic structure, the FFs used are deemed suitable for further analysis.

Since this study focuses on structural organization within DES–surfactant systems, we validated the microstructure of pure Glyceline and Reline by comparing RDFs obtained from classical MD and ab initio MD (AIMD) simulations. Thus, additional ab initio MD simulations were conducted, following the procedure of our previous work [4,109]. In brief, a pre-equilibrated box from classical MD, containing 10 DES units (10 choline cations, 10 chloride anions, and 20 HBD molecules), was used for AIMD with the QUICKSTEP module [110] of the CP2K 2023.2 software package [111]. The Becke–Lee–Yang–Parr (BLYP) functional [112,113], combined with a double-ζ valence polarized basis set (MOLOPT-DZVP-PBE-GTH) [114,115], was used for all atoms, while Goedecker–Teter–Hutter pseudopotentials [116] were applied to treat core electrons. Two short additional equilibration runs, each lasting 1 ps, were performed at elevated temperature (373.15 K) and then at 298.15 K to enhance sampling and stabilize the system. The main production run was then conducted at 298.15 K for 10 ps. During equilibration and production, the velocity Verlet algorithm was applied with time steps of 0.5 fs and 1 fs, respectively. All ab initio MD simulations used the NVT ensemble with a Nosé–Hoover thermostat [91,92] (50 fs time constant) to maintain a constant temperature, with periodic boundary conditions applied in all directions. The resulting trajectories were analyzed with TRAVIS to generate RDFs [117].

**Figure 2:**
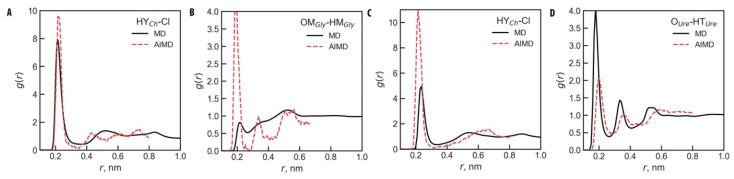
Radial distribution functions, *g*(*r*), between the choline cation and chloride anion (**A**) and (**C**) and HBD molecules (**B**) and (**D**), obtained from classic (black solid line) and ab initio MD (red dashed line) simulations in Glyceline (**A**) and (**B**) and Reline (**C**) and (**D**). For atom titles, see Figure 1A.

The key RDFs from ab initio MD and classical MD simulations are shown in Figure 2, with additional RDFs provided in Figure S2 of SI. As seen in Figure 2, the RDFs for the close contacts in Glyceline and Reline exhibit highly similar profiles between MD and AIMD: the overall shape of the curves is well aligned, with peaks and minima coinciding. This close match indicates that the structural representation achieved with the employed FFs is accurate and suitable for this study.

## 3. Results and Discussion

### 3.1. Free Energy of Solvation

As a first step of the analysis, the free energy of solvation, Δ*G*, was calculated for all surfactants in Ethaline, Glyceline, and Reline solvents. Free energy of solvation is a fundamental physical quantity that characterizes the transformation of a system from one state, gas phase in this case, to another state, the solvated state in this case.

The calculated free energies of solvation Δ*G* for the surfactants in the studied DESs, presented in Table 3, provide insights into their interactions and potential micelle-forming tendencies. Notably, all values are negative, indicating that the solvation processes are thermodynamically favorable across all systems examined. The variation in the magnitude of these negative Δ*G* values suggests differences in the strength of interactions between each surfactant and the DES components.

The least negative Δ*G* for SDS in Ethaline suggests relatively weaker interactions between SDS and Ethaline compared to other DES environments, implying that SDS in Ethaline experiences a comparatively less stabilized solvation. Conversely, SDS in both Glyceline and Reline shows slightly more negative Δ*G* values, indicating enhanced stabilization in these DESs relative to Ethaline. This stabilization may result from favorable interactions with the DES components that allow SDS to establish a stronger solvation shell.

CTAB in Glyceline displays a less negative Δ*G* than SDS in Glyceline, though still more negative than SDS in Ethaline, suggesting that CTAB experiences a relatively moderate stabilization in Glyceline. However, sulfobetaine surfactant, SB3-12, in Glyceline exhibits the most negative Δ*G*, exceeding twice that of CTAB in the same solvent, implying a highly favorable interaction between SB3-12 and Glyceline. This large negative value reflects strong interactions, possibly due to SB3-12’s compatibility with the hydrogen-bonded network within Glyceline, which could promote efficient solvation and micellization.

These results suggest that among the studied surfactants, SB3-12 in Glyceline is likely to exhibit the weakest tendency for micelle formation due to the substantial thermodynamic drive for solvation. Strong solvent–solute interactions favor solvation over micelle formation. In contrast, SDS demonstrates a greater tendency for micelle formation in both Glyceline and Reline, as does CTAB in Glyceline. SDS in Ethaline, with the least negative ΔG, may exhibit relatively more favorable micellization, reflecting weaker surfactant–solvent interactions. Overall, these findings underscore the significant influence of DES composition on the solvation behavior of each surfactant, with important implications for micelle stability and morphology in these unique solvents.

### 3.2. Shape and Radius

A fundamental aspect of micelle characterization involves examining how closely their structural configurations align with theoretical models. Theoretical representations often depict micelles as perfectly spherical aggregates, with monomer chains radially oriented towards the center. However, in practice, the extent to which micelles conform to this idealized shape and internal organization remains an open question, particularly in new and less-studied solvents such as DESs. It is therefore crucial to investigate whether surfactant molecules indeed assemble into near-perfect spherical structures and how their molecular chains orient themselves within the micelle.

As surfactant concentration increases, micelles tend to grow in size and may progressively deviate from spherical symmetry, transitioning into elongated or cylindrical forms. To quantify these shape variations, the eccentricity parameter, *e*, is employed, providing a direct measure of micelle sphericity. *e* is defined as follows: e=IminImean, where *I*_min_ is the smallest moment of inertia along the principal axes, and *I*_mean_ is the average moment of inertia across these axes. A value of *e* close to zero indicates a shape close to a perfect sphere, while increasing deviations from zero suggest more elongated or elliptical aggregates. Table 4 presents the eccentricity values for different micelles in DESs, obtained from MD simulations. These values provide insights into how micelle structures evolve as the surfactant concentration changes.

In Ethaline and Reline, the difference in eccentricity between small and large micelles is pronounced: large micelles exhibit significantly less spherical shapes, particularly in Reline. Notably, the large micelles, m120, in Ethaline are the least spherical among all the cases studied. This substantial difference in SDS micelle shape in Reline suggests that the concentration at which micelle elongation occurs is almost reached, and further increases in surfactant concentration could promote the formation of worm-like micelles. This observation aligns with findings from Arnold et al. [39], who experimentally demonstrated that SDS forms elongated structures in Reline.

In contrast to Ethaline and Reline, micelles in Glyceline become more spherical with increasing surfactant concentration, potentially due to stabilization by hydrogen bonds. Small micelles in Glyceline are already close to being perfectly spherical and become even more spherical with higher surfactant concentrations. The most spherical micelle among all systems is the small SB3-12 micelle in Glyceline, while the small SDS micelles have eccentricity values close to zero in both Reline and Glyceline. Interestingly, the larger SB3-12 and SDS micelles in Glyceline exhibit the same eccentricity of 0.07. Overall, large SDS micelles are most spherical in Glyceline. These findings are in line with previous experimental studies by A. Sanchez-Fernandez et al., who found that CTAB micelles are spherical [37] and SB3-12 micelles are globular [35] in Glyceline.

Among all SDS micelles, Ethaline promotes the greatest deviation from a spherical shape, whereas Reline results in the largest difference in shape between small and large micelles. This suggests that the solvent composition plays a critical role in determining micelle morphology, potentially affecting the aggregation dynamics and stability of surfactant assemblies in these DESs.

**Figure 3:**
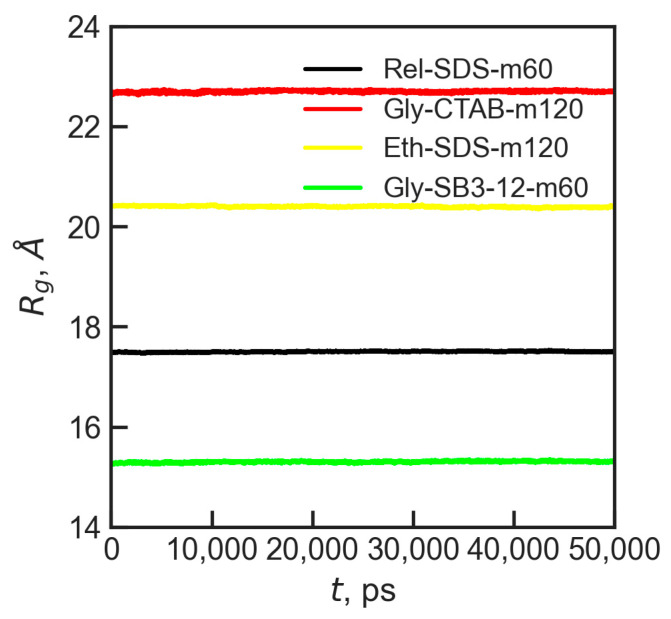
Change in *R*_g_ for selected micelles over a 50 ns production run in MD simulations.

To further assess the shape of the micelles, the radius of gyration, *R*_g_, was calculated as it is implemented in Gromacs [118]. The radius of gyration can be understood as the measure of the distance from a point around which a molecule rotates to the point where energy transfer is maximized. Additionally, calculating *R*_g_ can help confirm if the systems have reached equilibrium. The *R*_g_ exhibited similar behavior across all the studied systems, as illustrated by several examples shown in Figure 3. The plot in Figure 3, covering 50 ns of simulation time, confirmed that the micelles are equilibrated.

Based on the average radius of gyration, the mean effective micellar radius, *R*_s_, was calculated, following the approach of Palazzesi et al. [119], as Rs=53Rg. The effective radii, *R*_s_, and radii of gyration, *R*_g_, for all micelles studied are presented in Table 5.

The radius of gyration provides insight into the overall size and shape of the micelle, reflecting how far its components (i.e., surfactant molecules) are located from the center of mass. A larger *R*_g_ indicates a more dispersed mass distribution from the micelle’s center. In the studied systems, *R*_g_ is consistently larger for larger micelles (see Table 5), which is expected, with the largest value found for the CTAB-m120 micelle in Glyceline. A larger *R*_g_ suggests a less compact structure, which correlates with the observation that higher *R*_g_ values tend to be found for micelles with larger eccentricity (see Table 4). This observation is further supported by the similarity of *R*_g_ values for systems such as Gly-SDS-m60 and Gly-SB3-12-m60, Gly-SDS-m120, and Gly-SB3-12-m120, as well as Eth-SDS-m120 and Rel-SDS-m120, which also have similar eccentricity values. This suggests that these pairs of micelles have comparable shapes and similar interactions both internally and/or with the solvent.

Conversely, systems like Gly-BET-m60 and Eth-SDS-m60 show almost the same *R*_g_, while their eccentricity differs significantly (by a factor of two), indicating that other factors may be influencing the micelle rotation. Interestingly, all large SDS micelles exhibit similar *R*_g_ values in all DESs, whereas the small SDS micelles show noticeable differences in *R*_g_. This suggests that larger SDS micelles adopt a similarly compact structure regardless of the solvent. In contrast, smaller micelles likely exhibit more varied structures. This observation contradicts the finding of similar eccentricity values for smaller SDS micelles but differing values for larger ones. This inconsistency may relate to the internal structure of the micelles and the interactions between the micelles and the DESs.

Regarding effective radii, *R*_s_, Table 5 indicates that m60 micelles always have smaller radii than m120 micelles. However, the Gly-SB3-12-m120 micelle, with an *R*_s_ of 25.0 Å, is very similar in size to the Gly-CTAB-m60 micelle, which has an *R*_s_ of 24.3 Å, suggesting that Gly-BET-m120 is much more compact, while Gly-CTAB-m60 is less tightly packed. Among the small SDS micelles, those formed in Ethaline are the smallest, followed by those in Glyceline and Reline. In contrast, m120 micelles display similar radii across all systems.

In Glyceline, micelles formed by the zwitterionic surfactant SB3-12 are consistently smaller, possibly due to increased interactions within the micelle itself, which applies to both m60 and m120 micelles. CTAB, on the other hand, forms the largest micelles, which could be due to reduced interactions between CTAB and the solvent.

The effective radius of SDS in Reline was previously reported as approximately 17 Å [39], that of CTAB in Glyceline was reported as 28 Å [37], and that of SB3-12 in Glyceline as ~14 Å [28]. It should be noted that the radius of SDS in Reline was determined using scattering techniques and considered only the surfactant tails, as the headgroups were indistinguishable from the solvent based on their scattering lengths [39]. Given the scarcity of data and the complexity of the systems, the values we obtained are reasonably close to the experimental results. Comparing the sizes of SDS and CTAB micelles, it is known that CTA forms larger micelles than SDS [36], which is also in agreement with our findings.

For *R*_g_, there is considerable variation across experimental results. Reported values for *R*_g_ of SDS in Glyceline range from 16 Å [36] to approximately 18 Å [35], while in Reline, reported values are around 50 Å [36]. For CTAB in Glyceline, *R*_g_ ranges from 19–20 Å [38] to 26.1 Å [36,37]. Given these wide discrepancies in reported experimental values, our calculated *R*_g_ values seem to fall within the expected experimental range.

Due to the limited availability of simulated data on micelles in DESs, direct comparisons to other simulation studies are not possible. However, some data are available for MD simulations of SDS in water [48,54,55,62,120], where the reported radius for SDS micelles in water is approximately 21 Å, which aligns with our results. For SB3-12 micelles in water, simulation results range from 20 to 26 Å [63], while for CTAB, values range from 23 to 30 Å [63], depending on the force fields used for both water and the surfactants. Thus, when compared to simulation data, our findings are also consistent, especially considering the influence of the force fields used for both surfactants and DESs, which should be further explored in future studies.

### 3.3. Micelle Structure and Conformations

**Figure 4:**
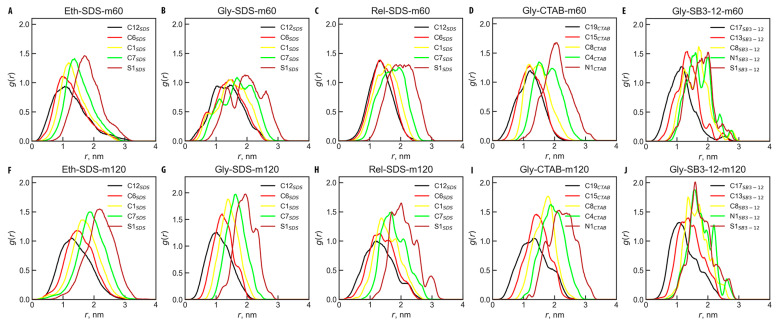
Radial distribution functions, *g*(*r*), between the center of the micelle and different atom types within the micelle. The systems and corresponding atoms are indicated in the figure for systems (**A**) Eth-SDS-m60; (**B**) Gly-SDS-m60; (**C**) Rel-SDS-m60; (**D**) Gly-CTAB-m60; (**E**) Gly-SB3-12-m60; (**F**) Eth-SDS-m120; (**G**) Gly-SDS-m120; (**H**) Rel-SDS-m120; (**I**) Gly-CTAB-m120; (**J**) Gly-SB3-12-m120; see Table 1 for system names.

Another approach to understanding the shape of micelles is by analyzing the radial distribution functions for specific atom types with respect to the center of mass (COM) of the micelle. The atom types considered were C12, C6, C1, C7, and S1 for SDS; C19, C15, C8, C4, and N1 for CTAB; and C17, C13, C8, N1, and S1 for SB3-12 (see Figure 1A for atom types). This analysis provides insight into the distribution of these atoms at specific distances from the micelle’s COM. Figure 4A–J show RDF graphs for all ten studied systems.

From the RDF analysis, it is evident that the atom distributions relative to the micelle’s COM are similar for both small and large micelles of the same kind, with larger micelles often showing smoother curves due to improved statistics. The SDS and CTAB micelles exhibit the typical arrangement of hydrophilic heads positioned towards the exterior and hydrophobic tails directed inward. As can be observed, for SB3-12 micelles, the longer alkyl radical (represented by the C17 atom; see Figure 1 for atom names) orients towards the micelle core, while the shorter alkyl tail (represented by the S1 atom) points outward. The terminal atoms—C12 for SDS, C19 for CTAB, and C17 for SB3-12—are found at the micelle center, with subsequent atoms along the alkyl chains positioned progressively outward, as expected.

**Figure 5:**
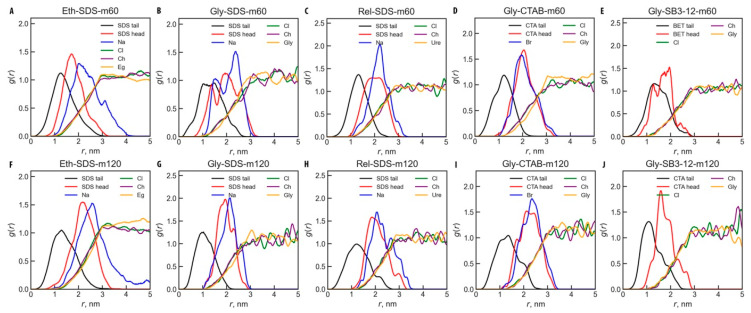
Radial distribution functions, *g*(*r*), between solvent ions and molecules and different parts of the micelles as a function of distance from the micelle’s COM. The corresponding atoms are indicated in the figure for systems (**A**) Eth-SDS-m60; (**B**) Gly-SDS-m60; (**C**) Rel-SDS-m60; (**D**) Gly-CTAB-m60; (**E**) Gly-SB3-12-m60; (**F**) Eth-SDS-m120; (**G**) Gly-SDS-m120; (**H**) Rel-SDS-m120; (**I**) Gly-CTAB-m120; (**J**) Gly-SB3-12-m120; see Table 1 for system names.

It is interesting to compare the distribution of different molecular segments (e.g., head and tail groups) and solvent components as a function of their distance from the micelle’s COM, as demonstrated in Figure 5. For this analysis, specific atoms were selected: C12 for the tail and S1 for the head group of SDS, along with the counter-ion Na^+^; C19 for the tail and N1 for the head group of CTAB, along with the counter-ion Br^−^; and C17 for the tail and S1 for the head of SB3-12. For DESs, we considered the COM of the choline cation, Ch^+^, the chloride anion, Cl^−^, and the HBD COM.

From Figure 5, it is observed that as the distance from the headgroup increases, the distribution of tail atoms broadens. Tail groups exhibit the largest distribution, extending up to 20–30 Å depending on micelle type and size, indicating that the tail atoms can exist anywhere between the COM and the micelle surface. Figure 1B,C show that headgroups do not entirely cover the micelle surface, allowing for solvent penetration into areas occupied by the alkyl tails. This is further evidenced by Figure 5, which shows an interfacial layer of 15–25 Å (depending on the micelle type and size), corresponding to the headgroup peaks. In this interfacial layer, solvent molecules can surround the headgroups and partially penetrate into the alkyl tail core, which remains solvent-free up to a radius of ~13 Å for smaller micelles and ~15 Å for larger ones. Importantly, all DES components—Ch^+^, Cl^−^, and HBD—begin interacting not with the deepest carbon atoms of the tails but with atoms beyond the headgroup. Thus, the micelles present a rough, hydrated surface layer of approximately 20 Å thickness, comprising headgroups, solvent ions, counter-ions (in the case of SDS and CTAB), and some hydrophobic tails, along with a hydrophobic core extending up to a radius of 15 Å.

Interestingly, both small and large CTAB micelles in Glyceline show a similar distribution for the counter-ion Br^−^ and the headgroup (Figure 5D,I), while SDS and Na^+^ exhibit a less superposed distribution in all DESs (Figure 5A–C,F–H).

Another method for assessing the micelle structure is by analyzing the conformation of individual surfactant molecules within the micelle. This was carried out by measuring the distance between the head and tail groups, as well as the angle formed between these two atoms and a central reference atom. For this analysis, we selected distances between N1 and C19 for CTAB, S1 and C12 for SDS, and S1 and C17 for SB3-12. To measure the bending angle, atoms C1 for SDS, C8 for CTAB, and C6 for SB3-12 were used (see Figure 1A for atom types). This approach allows us to understand the positioning of surfactant molecules within the micelle. The average distances and angles obtained are summarized in Table 6.

According to Table 6, SDS shows consistent head-to-tail distances of 14–14.5 Å and similar bending angles of 138–148 degrees, regardless of micelle size or solvent. This is consistent with previously reported bending of SDS surfactants observed in MD simulations of SDS–water systems [121,122]. CTAB micelles also exhibit consistent structural parameters across different micelle sizes, with head-to-tail distances of 17.7–17.8 Å and a bending angle of approximately 147 degrees, indicating that CTAB molecules adopt a slight but uniform bend in both large and small micelles.

SB3-12 micelles, on the other hand, show a distinctly different behavior: the tail-to-head distance is significantly smaller than expected for a rigid molecule, indicating that the surfactant molecules are significantly bent. This observation is supported by the S1-C6-C17 angle, which is closer to 90 degrees than to 180 degrees, highlighting the bent configuration. This is further confirmed by RDFs from Figure 4, where the S1 and N1 atoms (see Figure 1A for atom types) in SB3-12 are nearly superposed, indicating significant bending of these molecules.

### 3.4. Micelle–Solvent and Intra-Micellar Interaction

**Figure 6:**
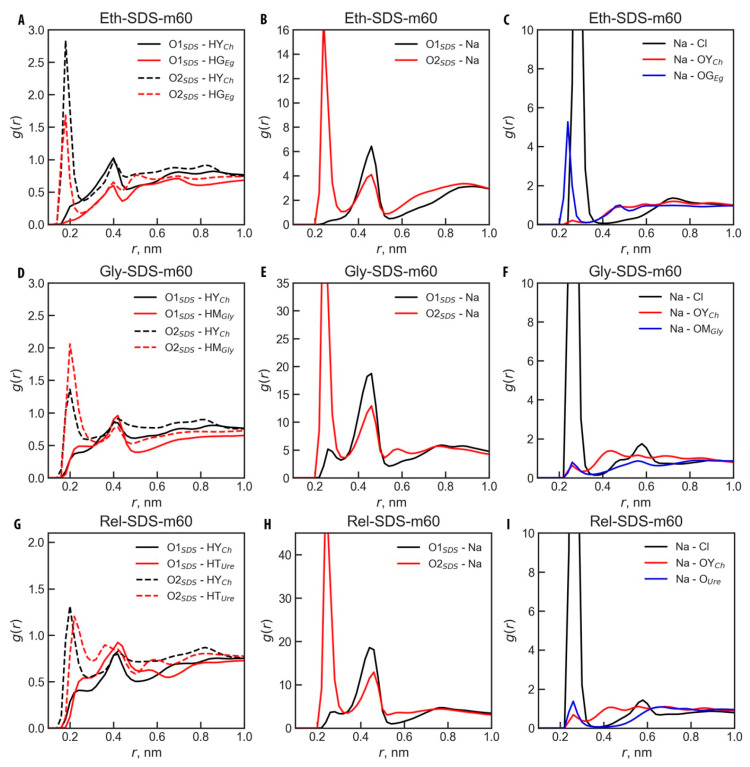
Radial distribution functions, *g*(*r*), for selected ion pairs in SDS-m60 micelle systems across all studied DESs. The pairs were chosen based on those exhibiting the highest absolute charge values. The corresponding atoms are indicated in the figure for systems (**A**–**C**) Eth-SDS-m60; (**D**–**F**) Gly-SDS-m60; (**G**–**I**) Rel-SDS-m60; see Table 1 for system names and Figure 1A for atom types.

**Figure 7:**
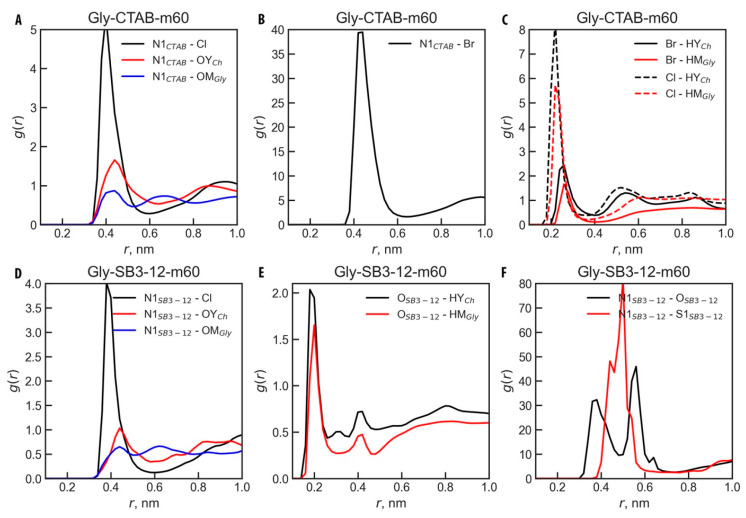
Radial distribution functions, *g*(*r*), for selected ion pairs in CTAB-m60 and SB3-12-m60 micelles in Glyceline. The pairs were chosen based on those exhibiting the highest absolute charge values. The corresponding atoms are indicated in the figure for systems (**A**–**C**) Gly-CTAB-m60; (**D**–**F**) Gly-SB3-12-m60; see Table 1 for system names and Figure 1A for atom types.

Further analysis of the molecular structure in all systems was conducted by calculating the RDFs between selected sites of each micelle and the active sites of the DES solvents. Figure 6 presents RDFs for SDS in all studied DESs, while Figure 7 shows RDFs for the Gly-SB3-12 and Gly-CTAB systems. The selected pairs involved atoms with the highest positively and negatively charged sites. It should be noted that Figure 6 and Figure 7, as well as the discussion below, only consider the small m60 micelles. However, the conclusions drawn are fully applicable to the larger m120 micelles, which exhibit virtually the same behavior (see Figures S3 and S4 in SI).

For all pairwise interactions involving SDS, the closest contacts consistently occurred between the oxygen atoms of SDS and the hydrogen atoms of the HBD component in each DES. This is particularly pronounced for the O2 atom of SDS, as the O2_SDS_–X (here, X is HG_Eg_, HM_Gly_, and HT_Ure_; see Figure 6A,D,G) interaction peaks at ~0.2 nm across all DESs, whereas the O1_SDS_–X interaction appears at ~0.4 nm due to the geometric position of the O1_SDS_ atom (see Figure 6A,D,G). In Ethaline, the O2_SDS_ atom interacts more intensely with the choline cation Ch^+^ than with ethylene glycol, though both interactions (O2_SDS_–HY_Ch_ and O2_SDS_–HG_Eg_) peak at 0.18 nm (see Figure 6A). In Glyceline, O2_SDS_ interacts more intensively with the hydrogen atoms of Gly, compared to Ch^+^, with an interaction distance of 0.2 nm (see Figure 6D). In Reline, the O2_SDS_–HY_Ch_ and O2_SDS_–HT_Ure_ interactions occur at slightly different distances, with the cation able to approach SDS more closely, peaking at 0.2 nm, compared to 0.22 nm for O2_SDS_–HT_Ure_ (see Figure 6G).

The Na^+^ counter-ion interacts preferentially with the O2_SDS_, with a consistent RDF peak at 0.24 nm across all DESs (Figure 6B,E,H). Although these interactions are intense, the choline cation and the HBD can approach the O2 of SDS more closely, suggesting the formation of hydrogen bonds. In Ethaline, Na^+^ also interacts significantly with OG_Eg_ (see Figure 6C), potentially disrupting the Hbond network, whereas such interactions are negligible in other solvents.

In the Gly-CTAB-m60 system, the only significant short-distance interactions are registered between Cl^−^ or Br^−^ anions and the active hydrogens from Ch^+^ and Gly (Figure 7C). The quaternary ammonium group of CTAB carries a positive charge, which is effectively screened by methyl and cetyl groups. Consequently, there are no strongly charged atoms readily accessible for interactions, as evidenced by RDF peaks at relatively long distances (~0.4 nm, Figure 7A). Despite the long distance, the N1–Cl and N1–Br interactions are pronounced, with the N1–Br interaction being significantly more intense (Figure 7A,B), which is noteworthy given the smaller number of Br^−^ ions in the system compared to Cl^−^ (see Table 1 for systems composition). Additionally, Br^−^ and Cl^−^ interact intensively at short distances with Ch^+^ and Gly HBD, with Cl^−^ interacting more closely, as indicated by RDF peaks for Cl–HY_Ch_ and Cl–HM_Gly_ at 0.22 nm, while Br–HY_Ch_ and Br–HM_Gly_ appear at 0.26 nm (Figure 7C). These observations suggest that the CTA surfactant perturbs the DES structure less significantly compared to SDS.

In the Gly-SB3-12-m60 system, SB3-12, as a zwitterionic surfactant, exhibits distinct interaction patterns. Due to its charge balance, SB3-12 is prone to self-interaction in addition to its interactions with the solvent. The ammonium group of SB3-12 can interact with all Glyceline components, but, similar to CTAB, its charge is partially shielded by alkyl groups, resulting in interactions occurring at longer distances (~0.4 nm, Figure 7D,F). These interactions are more pronounced for Cl^−^, which carries the most localized negative charge. In contrast, interactions involving the accessible oxygen atom in SB3-12 occur at shorter distances, with well-defined RDF peaks: O_SB3-12_–HY_Ch_ at 0.18 nm and O_SB3-12_–HM_Gly_ at 0.20 nm (Figure 7E), suggesting possible hydrogen bond formation. Self-interactions between SB3-12 molecules are evident from Figure 7F, which shows distinct RDF peaks for N1_SB3-12_–O_SB3-12_ and N1_SB3-12_–S1_SB3-12_ interactions. Although these occur at long distances, they reflect the internal interactions of hidden atoms attached to hydrogens capable of hydrogen bonding.

Consistent with previous studies [4,6,13,109], the Luzar and Chandler [123] criterion was adopted for Hbond analysis. According to this criterion, the donor–acceptor distance and the H···donor–acceptor angle cut-offs were set at 0.35 nm and 30°, respectively. Given the significant role of Hbonds in DESs, these interactions can either disrupt or modify the presence of micelles. Based on the RDF analysis, Hbonds are expected in systems with SDS (between the sulfate group of SDS and DES active sites), SB3-12 (involving the same group and DES active sites), and potentially from the ammonium groups of SB3-12 and CTAB. The present ions—Br^−^ and Na^+^—may also influence the Hbonding network. The results of the Hbond analysis are presented in Table 7, considering all species as both donors and acceptors.

Table 7 shows that the number of Hbonds is proportional to micelle size, with larger micelles generally forming ~50% to 100% more Hbonds. The largest increase, nearly twofold, is observed for SB3-12 micelles in Glyceline (286 Hbonds for m60 versus 568 for m120), followed by a 91% increase for SDS micelles in Ethaline (64 Hbonds for m60 versus 122 for m120). For the remaining systems, the increase in the number of Hbonds is less pronounced, ranging from 47% to 61%.

Overall, the number of Hbonds involving SDS represents only 2–5% of all Hbonds in the studied systems. SB3-12 forms significantly more connections, accounting for 7.7% of all Hbonds in systems with small micelles and 14.2% in systems with large micelles. In contrast, CTAB micelles form the fewest number of Hbonds, contributing only ~1.5% in Gly-CTAB-m60 and ~2.3% in Gly-CTAB-m120.

SDS primarily forms Hbonds with HBD, independently of the DES, followed by Ch^+^. In Glyceline and Reline, the number of Hbonds formed between SDS and HBD is two to three times greater than those formed with Ch^+^. SDS almost does not form Hbonds with itself or with Cl^−^. CTAB, overall, forms fewer Hbonds, and the distribution differs from that of SDS: CTAB does not form Hbonds with itself or with Ch^+^, while the number of Hbonds formed between CTAB and Cl^−^ or CTAB and Ure is almost identical, regardless of micelle size. This observation aligns well with a previously reported study [36], which found that the headgroup of the SDS anionic surfactant is primarily solvated by HBD species in a ternary DES consisting of choline chloride, urea, and glycerol. In contrast, CTAB exhibits significant solvent–headgroup interactions only at higher glycerol concentrations.

SB3-12 appears to be the most prone to Hbond formation, forming significantly more Hbonds than SDS or CTAB, and with all species present in the system. Notably, the largest contributors to Hbonds involving SB3-12 are the HBD (Gly) and SB3-12 itself, with more than half of the Hbonds involving SB3-12 being formed between SB3-12 molecules. These findings align with the RDF analysis results (Figure 6 and Figure 7): CTAB did not show potential for Hbonding with Ch^+^ and HBD, interacting mainly through Cl^−^. SDS displayed moderate potential for Hbonding with HBD and Ch^+^, while SB3-12 showed strong interactions with DES components, particularly through well-defined O_SB3-12_–HY_Ch_ and O_SB3-12_–HM_Gly_ peaks, as well as significant SB3-12–SB3-12 interactions.

Regarding the influence of micelle presence on the Hbonding network inherent in DESs, we compared the percentage of Hbonds formed between DES species in this study (see Table S1, SI) with simulated data from the literature [4,6,13,109]. Since no data on Hbonding in Reline were available, we conducted an analysis of pure Reline, with results presented in Table S1. Importantly, with six Glyceline systems analyzed, the overall distribution of Hbonds was consistent, with only 1–2% variation, indicating good system stability.

As seen in Table S1, the overall distribution of different types of Hbonds in systems with small and large micelles is very similar, with micelle size having little effect. While there are minor variations in the number and percentage of certain interactions, these are statistically insignificant. Interestingly, in all systems except Ethaline, the total number of Hbonds formed exclusively between DES components is slightly higher in systems with m120 micelles. This trend is most pronounced in Reline, where the primary contributor to the increase is HBD–HBD Hbonds. The percentage of Ch–Ch Hbonds in pure Ethaline and Glyceline is minimal, contributing only up to 1% (and 2% in Reline). In systems with micelles, this percentage increases slightly in Ethaline and Glyceline, reaching 1.1–1.6%, while decreasing to 1.9% in Reline. In pure DESs, Ch–HBD and Ch–Cl Hbonds are least prevalent in Glyceline, each comprising only 8% of total Hbonds, compared to 13–22% in Ethaline.

In the systems with micelles, the distribution of Hbonds between DES components differs slightly (Table S2, SI). In all Glyceline systems, the interactions involving Ch–HBD and Ch–Cl constitute more than 8% of the total Hbonds, with the percentage of Ch–Cl Hbonds consistently slightly higher by around 1–2%. The highest percentage of H-bonds is seen for Cl–HBD interactions in Glyceline (45%) and Reline (41%). This aligns well with our findings, where the Cl–HBD percentage in Glyceline fluctuates between 47.2% and 49.5%, and that of Reline fluctuates between 41% and 42%. HBD–HBD interactions were reported as the most significant in Ethaline and Glyceline, accounting for 38–49% and 39% of Hbonds, respectively. Our findings are partly consistent with these observations: while HBD–HBD interactions in Ethaline account for ~45%, in Glyceline, this percentage is significantly lower, at around 28–30%, similar to Reline (~29%). This decrease in HBD–HBD interactions in Glyceline is likely due to Hbonds forming between HBDs and surfactant molecules in the Glyceline environment.

Despite some localized perturbations caused by the presence of micelles, our results indicate that micelles do not significantly disrupt the hydrogen bonding network inherent in DESs. This suggests that the Hbonding within the solvent remains largely intact, maintaining the bulk properties of the DES even in the presence of self-assembled surfactant structures. This observation is consistent with earlier findings by Atri et al. [36], which indicate that differences in micelle morphology are not driven by variations in hydrogen bonding between solvent components.

### 3.5. Transport Properties

**Figure 8:**
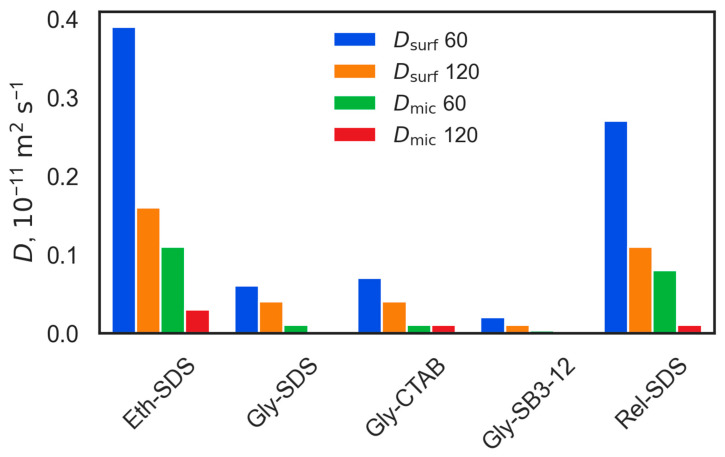
Self-diffusion coefficients, *D*, for individual surfactants and m60 and m120 micelles across all DESs studied here, obtained from MD simulations at 298.15 K and 1 bar.

Determining dynamic properties is crucial for understanding transport mechanisms within these systems, offering insights into ion and molecular mobility as well as the structural stability of the systems. However, obtaining precise dynamic properties through MD simulations is challenging, particularly for viscous liquids such as DESs [6,124]. The accuracy of these calculations depends on several factors, including simulation time, sample size, the force field model used, and the specific methodology applied. Consequently, it is virtually impossible to achieve highly accurate results for the dynamic properties of the solutions studied here. The diffusion coefficient, *D*, values presented in Table S3 (for all species present in the studied systems) and Figure 8 (for individual surfactants, *D*_surf_, and micelles, *D*_mic_, in systems with small (60) and large (120) micelles) are thus rather qualitative indicators of the dynamics in the studied systems.

All diffusion coefficients are lower than their experimental counterparts, as expected, since no special techniques (e.g., charge scaling) were used to enhance the dynamics. Nevertheless, as shown in Table S3, the diffusion coefficients for Ethaline components (i.e., Ch, Cl, and Eg) are in closer agreement with experimental results [106,109] and previously reported simulations [4,6,109]. This can be attributed to the fact that the FF model for Ethaline was specifically developed for a range of temperatures, whereas the FFs for the other DESs are only known to perform well at higher temperatures [80].

The diffusion coefficients for all DES species are lower compared to reported simulations of pure DESs. This is unsurprising, given that the presence of large micelles disrupts the overall dynamics. Importantly, the order of species diffusion remains consistent, particularly for *D*_Ch_ and *D*_HBD_. The diffusion coefficients for Ch and HBD can be compared to experimental values, while *D*_Cl_ can only be compared with simulation values for Ethaline and Glyceline. Specifically, in Ethaline, the diffusion of the HBD species is consistently larger than that of Cl, which in turn is larger than that of Ch, as previously described [106,109]. In Glyceline, HBD diffuses more readily than Ch, consistent with experimental findings [106]. In Reline, the diffusion of the HBD is roughly twice that of Ch, which also aligns with experimental data [106].

Interestingly, diffusion coefficients for all DES species are consistently higher in systems with small m60 micelles compared to systems with larger aggregates. This is likely due to the greater surface-area-to-volume ratio of smaller micelles, which enhances interaction between DES molecules and micelle surfaces without extensive structuring. In contrast, larger micelles create more structured, less dynamic solvation layers that restrict DES mobility, thereby lowering diffusion rates.

For Ch^+^ in Glyceline, diffusion remains almost unchanged across all systems. The diffusion coefficient for HBD is highest in the CTAB system, possibly due to the fewer hydrogen bonds formed between micelles and the solvent in this system. In Glyceline, the diffusion of Cl^−^ is lowest, likely due to the strong interaction between Cl^−^ and additional Na^+^ cations (see Figure 6B,C,H). However, in CTAB systems, Cl^−^ diffusion is higher, probably due to competition from Br- anions for interaction sites, which reduces the extent of interactions involving Cl^−^ (see Figure 7C).

The diffusion coefficients for individual surfactants and micelles are presented in Figure 8. Unfortunately, no experimental values are available for surfactant–DES systems. However, in comparison to the experimental diffusion coefficients for SDS and CTAB in water (26·10^−11^ m^2^ s^−1^ and 20·10^−11^ m^2^ s^−1^, respectively) [125,126], the diffusion coefficients for SDS and CTAB in Glyceline—despite being much smaller—are still relatively close to one another. In all studied systems, individual surfactant molecules diffuse significantly faster than micelles. Consistent with the trend for DES components, the diffusion of surfactants and micelles is higher in systems with m60 micelles, due to fewer local perturbations compared to systems with larger aggregates. As expected, the smaller micelles exhibit significantly higher diffusion coefficients than the larger ones.

The mobility of SDS surfactants and micelles is greater in Ethaline and Reline, which have less dense Hbonding networks compared to Glyceline. In Glyceline, CTAB is the most mobile species, which can be explained by the fewer hydrogen bonds formed between CTAB and the DES. In contrast, SB3-12 exhibits the lowest diffusion coefficient, reflecting the strong hydrogen bonding between SB3-12, itself, and the solvent, particularly with Ch^+^ and the HBD.

## 4. Conclusions

In this study, we conducted molecular dynamics simulations to explore the structure, dynamics, and interactions of sodium dodecyl sulfate (SDS), cetyltrimethylammonium bromide (CTAB), and N-dodecyl-N,N-dimethyl-3-ammonio-1-propanesulfonate (SB3-12) micelles in three deep eutectic solvents: Ethaline, Glyceline, and Reline. Our findings reveal several key insights into micelle behavior and interactions in these solvents, highlighting the role of solvent composition in influencing micellar properties.

The free energy of solvation indicated favorable solvation processes for all surfactants. Structural analysis, including eccentricity and radius of gyration, showed that micelle morphology strongly depends on the DES used. In Ethaline and Reline, large micelles were less spherical, with SDS in Ethaline showing the greatest deviation, suggesting potential worm-like micelle formation. In contrast, micelles in Glyceline, particularly those formed by SB3-12, tended to become more spherical and compact with increased surfactant concentration, likely due to hydrogen bond stabilization. These trends were consistent with experimental data.

Larger micelles generally had greater radii and less compact structures, while SB3-12 micelles in Glyceline were more spherical and compact. This suggests that micelle compactness is influenced by solvent–surfactant interactions. This behavior aligns with the observed differences in effective radii and micelle rigidity, suggesting that micelle compactness is influenced by the balance of intra- and inter-molecular forces in DESs.

Radial distribution functions revealed notable differences in micelle–solvent interactions: SDS interacted closely with HBD components, while CTAB showed fewer interactions due to its screened ammonium group. SB3-12 exhibited strong self-interaction and significant interactions with DES components, indicating that DES composition significantly influences micelle–solvent interactions.

Hbond analysis confirmed that micelles minimally perturb the DES Hbonding network. SB3-12 contributed more significantly to Hbonding compared to SDS and CTAB, while CTAB formed the fewest Hbonds, suggesting limited influence on the DES structure. The micelles formed in Glyceline showed a consistent Hbond distribution, indicating a stable system even with surfactant structures.

The evaluation of transport properties highlighted that diffusion coefficients were consistently lower in systems with larger micelles, attributed to more local structural perturbations restricting mobility. The presence of smaller micelles led to higher diffusion coefficients for DES components and surfactants. SDS and CTAB showed comparable diffusion rates in Glyceline, while SB3-12 had the lowest mobility due to its strong Hbonding interactions.

Summarizing, this study provides insights into the structural and dynamic properties of micelles in DESs, emphasizing the critical role of solvent composition in determining micelle shape, interactions, and mobility. These findings contribute to optimizing DES–surfactant systems for applications such as electrodeposition, nanomaterial templating, and drug delivery, where precise control over micellar properties is essential. Future studies will focus on investigating the interactions of surfactants with surfaces, which is crucial for optimizing these applications.

## Figures and Tables

**Table 1:** Composition of the studied systems.

System Name	DES Name	HBD	Surfactant	Number of Surfactants in the Micelle	Total Number of Interaction Sites	Final Simulation Box Length, nm
Eth-SDS-m60	Ethaline	Eg	SDS	60	45,588	7.745
Gly-CTAB-m60	Glyceline	Gly	CTAB	60	54,980	7.978
Gly-SB3-12-m60	Glyceline	Gly	SB3-12	60	54,740	7.922
Gly-SDS-m60	Glyceline	Gly	SDS	60	53,780	7.904
Rel-SDS-m60	Reline	Ure	SDS	60	41,492	7.546
Eth-SDS-m120	Ethaline	Eg	SDS	120	48,168	7.893
Gly-CTAB-m120	Glyceline	Gly	CTAB	120	58,760	8.132
Gly-SB3-12-m120	Glyceline	Gly	SB3-12	120	58,280	8.064
Gly-SDS-m120	Glyceline	Gly	SDS	120	56,360	8.028
Rel-SDS-m120	Reline	Ure	SDS	120	44,072	7.577

**Table 2:** Densities, *d*, and self-diffusion coefficients, *D*, in the simulated pure Glyceline and Reline at 298.15 K. Experimental values from the literature are provided for comparison. Standard deviations are presented in parentheses.

DES	*d*, kg m^−3^			*D*, 10^−11^ m^2^ s^−1^					
				Choline Cation			HBD		
	Sim	Exp	Error, %	Sim	Exp	Error, %	Sim	Exp	Error, %
Glyceline	1183.7 (0.1)	1180 [2]	0.3	0.17 (0.01)	0.38 [2]	55	0.30 (0.01)	0.52 [2]	42
		1192 [103]	0.7						
		1191.2 [104]	0.6						
Reline	1135.6 (0.2)	1240 [2]	8	* 0.52 (0.03)	0.35 [2]		* 0.94 (0.03)	0.66 [2]	
		1196.55 [105]	4						
		1198 [80]	5						

* Values obtained at 373.15 K.

**Table 3:** Free energy of solvation, Δ*G*, of individual surfactants in the studied DESs, obtained from MD simulations at 298.15 K and 1 bar. Standard deviations are provided in parentheses.

DES	Surfactant	Δ*G*, kJ mol^−1^
Ethaline	SDS	−21 (3)
Glyceline	SDS	−36 (1)
	CTAB	−27.6 (0.7)
	SB3-12	−59 (1)
Reline	SDS	−32.0 (0.4)

**Table 4:** Eccentricity values, *e*, for various micelles in different DESs, obtained from MD simulations at 298.15 K and 1 bar.

	Ethaline	Glyceline	Reline
SDS-m60	0.114	0.09	0.066
CTAB-m60		0.12	
SB3-12-m60		0.05	
SDS-m120	0.202	0.07	0.134
CTAB-m120		0.107	
SB3-12-m120		0.07	

**Table 5:** Effective radii, *R*_s_, and radii of gyration, *R*_g_, for micelles in Reline, Glyceline, and Ethaline.

Micelle	Ethaline	Glyceline	Reline	Ethaline	Glyceline	Reline
	*R*_s_, Å			*R*_g_, Å		
SDS-m60	19.9	21.0	22.6	15.4	16.2	17.5
CTAB-m60		24.3			18.8	
SB3-12-m60		19.7			15.3	
SDS-m120	26.3	26.0	25.8	20.4	20.1	20.0
CTAB-m120		29.3			22.7	
SB3-12-m120		25.0			19.4	

**Table 6:** Average head-to-tail distances and bending angles for studied micelles systems, obtained from MD simulations at 298.15 K and 1 bar. Standard deviations are presented in parentheses.

System	Atoms Forming Head–Tail Distance	Distance, Å	Atoms Forming Angle	Angle, Degrees
Eth-SDS-m60	S1-C12	14.1 (0.2)	S1-C1-C12	143 (2)
Eth-SDS-m120		14.5 (0.1)		148 (1)
Gly-SDS-m60	S1-C12	14.0 (0.1)	S1-C1-C12	142 (2)
Gly-SDS-m120		14.3 (0.1)		146 (2)
Gly-SB3-12-m60	S1-C17	9.9 (0.2)	S1-C6-C17	118 (2)
Gly-SB3-12-m120		9.9 (0.1)		119 (1)
Gly-CTAB-m60	N1-C19	17.7 (0.2)	N1-C8-C19	146 (2)
Gly-CTAB-m120		17.8 (0.2)		148 (3)
Rel-SDS-m60	S1-C12	13.7 (0.2)	S1-C1-C12	138 (3)
Rel-SDS-m120		14.2 (0.1)		143 (2)

**Table 7:** Average number of hydrogen bonds and their relative contribution derived from MD simulation at 298.15 K and 1 bar. Standard deviations are presented in parentheses.

System	Hbonds	Hbonds Involving Surfactant				Hbond Involving Only DES Components					Total
		Surf–Surf	Surf–Ch	Surf–HBD	Surf–Cl	Ch–Ch	Ch–HBD	Ch–Cl	Cl–HBD	HBD–HBD	
Eth-SDS- m60	Number	1 (1)	27 (5)	36 (5)	0	33 (8)	591 (1)	313 (14)	727 (18)	1373 (47)	3101
	%	0.0	0.9	1.2	0.0	1.1	19.1	10.1	23.4	44.3	100
		2.1				97.9					
Eth-SDS- m120	Number	2 (2)	54 (7)	66 (7)	0	32 (8)	583 (21)	302 (15)	704 (21)	1375 (53)	3118
	%	0.1	1.7	2.1	0.0	1.0	18.7	9.7	22.6	44.1	100
		3.9				96.1					
Gly-SDS- m60	Number	0.6 (1)	19 (4)	70 (7)	0	52 (11)	328 (19)	383 (18)	1584 (30)	1012 (42)	3449
	%	0.0	0.6	2.0	0.0	1.5	9.5	11.1	45.9	29.3	100
		2.6				97.4					
Gly-SDS-m120	Number	3 (2)	41 (6)	98 (8)	0	55 (10)	350 (17)	359 (14)	1616 (34)	1006 (43)	3528
	%	0.1	1.2	2.8	0.0	1.6	9.9	10.2	45.8	28.5	100
		4.0				96.0					
Gly-SB3-12-m60	Number	163 (17)	24 (4)	75 (7)	24 (4)	54 (11)	320 (18)	390 (15)	1650 (29)	1003 (42)	3703
	%	4.4	0.6	2.0	0.6	1.5	8.6	10.5	44.6	27.1	100
		7.7				92.3					
Gly-SB3-12-m120	Number	352 (28)	36 (5)	145 (11)	35 (5)	55 (10)	332 (19)	378 (15)	1695 (38)	967 (45)	3995
	%	8.8	0.9	3.6	0.9	1.4	8.3	9.5	42.4	24.2	100
		14.2				85.8					
Gly-CTAB-m60	Number	0.4 (0.8)	5 (2)	21 (4)	25 (5)	50 (10)	316 (17)	382 (15)	1649 (35)	1013 (46)	3461
	%	0.0	0.1	0.6	0.7	1.4	9.1	11.0	47.6	29.3	100
		1.5				98.5					
Gly-CTAB-m120	Number	1 (2)	8 (3)	36 (6)	37 (6)	55 (11)	343 (18)	370 (17)	1711 (60)	979 (42)	3540
	%	0.0	0.2	1.0	1.0	1.6	9.7	10.5	48.3	27.7	100
		2.3				97.7					
Rel-SDS- m60	Number	0.6 (1)	21 (5)	76 (10)	0	50 (10)	360 (21)	386 (15)	1121 (29)	774 (39)	2789
	%	0.0	0.8	2.7	0.0	1.8	12.9	13.8	40.2	27.8	100
		3.5				96.5					
Rel-SDS- m120	Number	3 (2)	36 (6)	104 (10)	0	53 (10)	396 (19)	378 (25)	1167 (71)	838 (49)	2975
	%	0.1	1.2	3.5	0.0	1.8	13.3	12.7	39.2	28.2	100
		4.8				95.2					

## Data Availability

The data presented in this study are available upon request from the corresponding author, as the large volume of data precludes direct public sharing.

## Supplementary Materials

The following supporting information can be downloaded at: https://www.mdpi.com/article/10.3390/molecules30030574/s1. Figure S1. Beta, *β*, vs. time for Reline at 298 K and 373 K and Glyceline at 298 K derived from MD simulations at 1 bar. Figure S2. Radial distribution functions, *g*(*r*), between choline cations, choline cation and HBD molecules, chloride anion and HBD molecules, and between HBD molecules themselves obtained from classic and ab initio MD simulations in Glyceline and Reline. Figure S3. Radial distribution functions, *g*(*r*), for selected ion pairs in SDS-m120 micelle systems across all studied DESs. Figure S4. Radial distribution functions, *g*(*r*), for selected ion pairs in CTAB-m120 and SB3-12-m120 micelles in Glyceline. Table S1. Average relative contributions of Hbonds in pure DESs derived from MD simulation at 298.15 K and 1 bar in this work and from the literature. Table S2. Average relative contributions of Hbonds in DES–surfactant systems, considering only hydrogen bonds involving DES components, derived from MD simulation at 298.15 K and 1 bar. Table S3. Diffusion coefficients derived in this study from MD simulations at 298.15 K and 1 bar.
