# Supplementary material for: Influence of Deep Eutectic Solvent Composition on Micelle Properties: A Molecular Dynamics Study
Source: Molecules. 2025 Jan 27;30(3):574. doi: 10.3390/molecules30030574 (PMC11821091; doi:10.3390/molecules30030574)
Supplement: Supplementary file 1 [file molecules-30-00574-s001.zip › molecules-3416917-supplementary.pdf]

Influence of Deep Eutectic Solvent Composition on Micelle Properties: A Molecular Dynamics Study

Iuliia V. Voroshylova, Elisabete S. C. Ferreira, M. Natália D. S. Cordeiro

REQUIMTE LAQV, Department of Chemistry and Biochemistry, Faculty of Sciences, University of Porto, 4169-007 Porto, Portugal
* Correspondence: voroshylova.iuliia@fc.up.pt

Supporting information

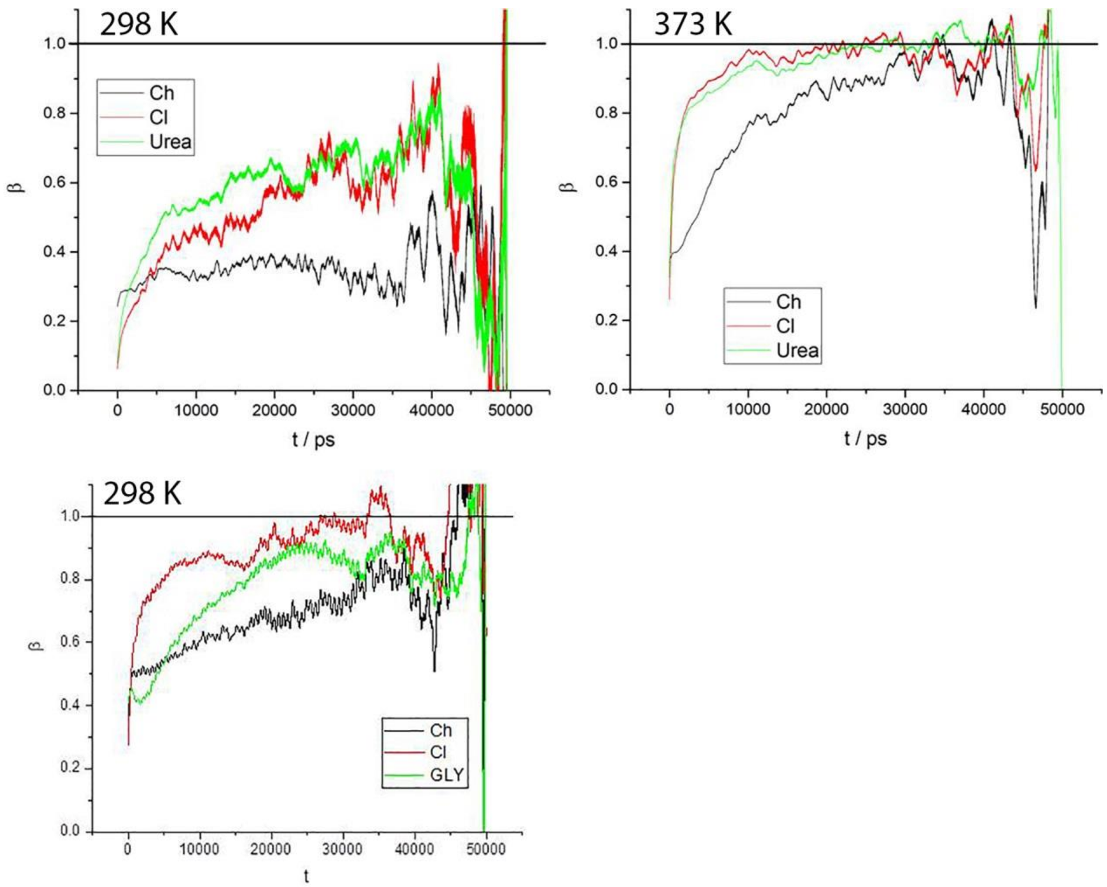

Figure S1. Beta, β, vs time for Reline at 298 K (top left) and 373 K (top right) and Glyceline at 298 K (bottom). The curves for Ch^+ cation are black, for Cl^- anion are red and for HBD are green. The data was derived from MD simulations at 1 bar.

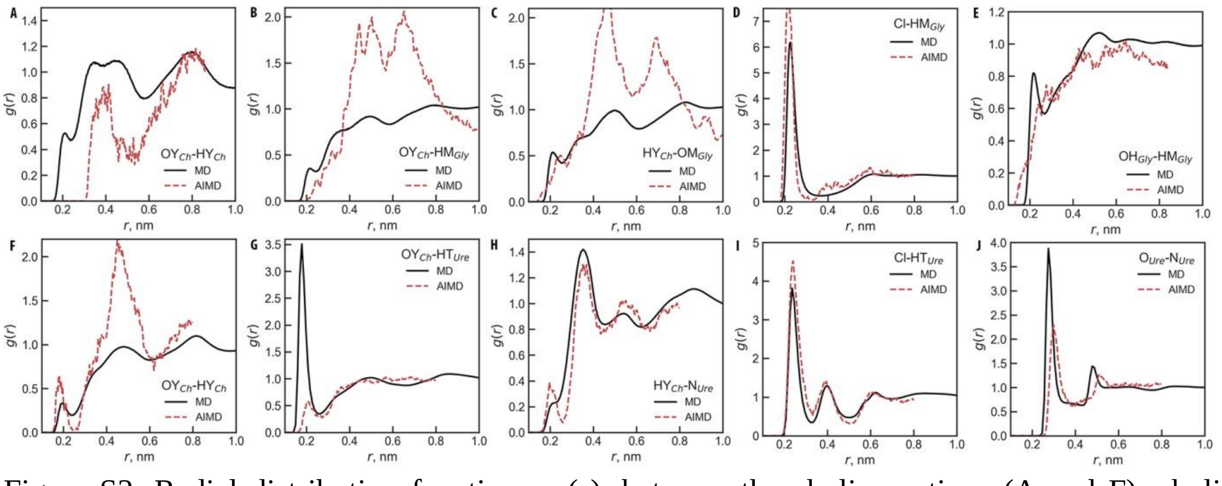

Figure S2. Radial distribution functions, $g(r)$, between the choline cations (A and F), choline cation and HBD molecules (B, C, G and H), chloride anion and HBD molecules (D and I), and between HBD molecules themselves (E and J) obtained from classic (black solid line) and ab initio (red dashed line) MD simulations in Glyceline (top line) and Reline (bottom line). For atom titles consult Figure 1.

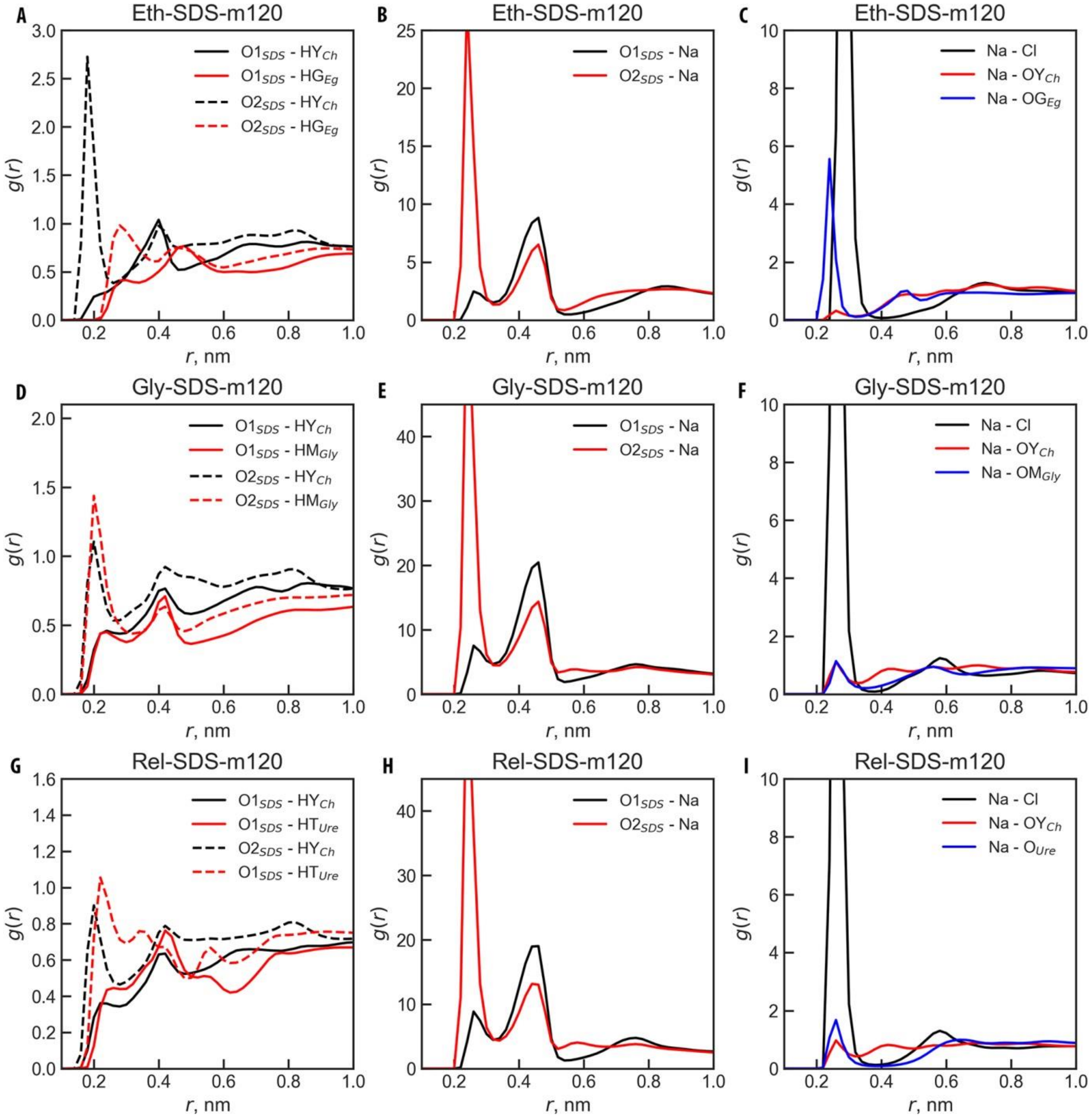

Figure S3. Radial distribution functions, $g(r)$, for selected ion pairs in SDS-m120 micelle systems across all studied DESs. The pairs were chosen based on those exhibiting the highest absolute charge values. The systems and corresponding atoms are indicated in the figure, consult Table 1 for systems names and Figure 1 for atom types.

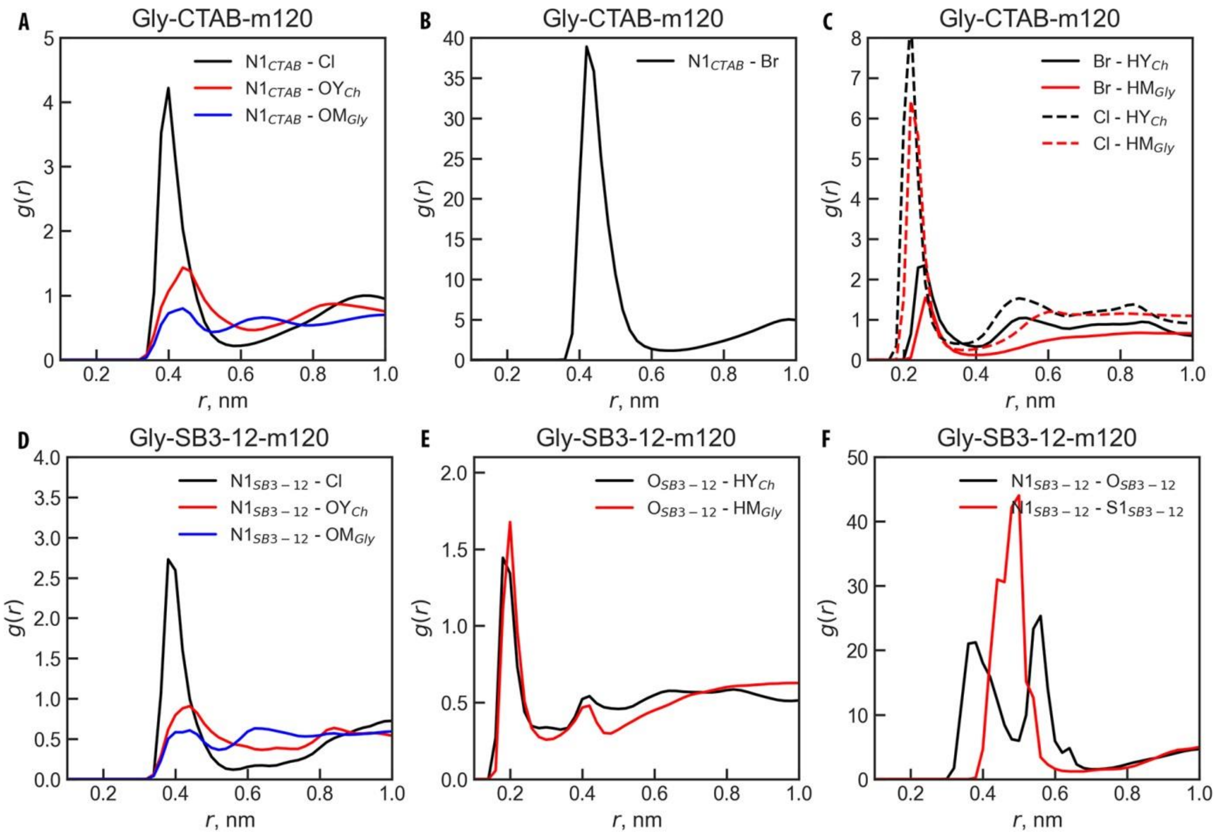

Figure S4. Radial distribution functions, $g(r)$, for selected ion pairs in CTAB-m120 and SB3-12-m120 micelles in Glyceline. The pairs were chosen based on those exhibiting the highest absolute charge values. The systems and corresponding atoms are indicated in the figure, consult Table 1 for systems names and Figure 1 for atom types.

Table S1. Average relative contributions of Hbonds in pure DESs derived from MD simulation at 298.15 K and 1 bar in this work and from literature

	Ethaline [1]	Ethaline [2]	Ethaline [3]	Glyceline [2]	Reline, this work
Ch-Ch	0.2	0	1	0	2
Ch-HBD	13.6	16	22	8	15
Ch-Cl	12.8	14	12	8	13
Cl-HBD	24.8	32	24	45	41
HBD-HBD	48.6	38	40	39	29

Table S2. Average relative contributions of Hbonds in DES-surfactant systems, considering only hydrogen bonds involving DES components, derived from MD simulation at 298.15 K and 1 Bar. See Table 1 for systems' names.

	Ch-Ch	Ch-HBD	Ch-Cl	Cl-HBD	HBD-HBD	Total
Eth-SDS-m60	33	591	313	727	1373	3037
% in DES	1.1	19.5	10.3	23.9	45.2	100
Eth-SDS-m120	32	583	302	704	1375	2996
% in DES	1.1	19.5	10.1	23.5	45.9	100
Gly-SDS-m60	52	328	383	1584	1012	3359
% in DES	1.5	9.8	11.4	47.2	30.1	100
Gly-SDS-m120	55	350	359	1616	1006	3386
% in DES	1.6	10.3	10.6	47.7	29.7	100
Gly-BET-m60	54	320	390	1650	1003	3417
% in DES	1.6	9.4	11.4	48.3	29.4	100
Gly-BET-m120	55	332	378	1695	967	3427
% in DES	1.6	9.7	11.0	49.5	28.2	100
Gly-CTA-m60	50	316	382	1649	1013	3410
% in DES	1.5	9.3	11.2	48.4	29.7	100
Gly-CTA-m120	55	343	370	1711	979	3458
% in DES	1.6	9.9	10.7	49.5	28.3	100
Rel-SDS-m60	50	360	386	1121	774	2691
% in DES	1.9	13.4	14.3	41.7	28.8	100
Rel-SDS-m120	53	396	378	1167	838	2832
% in DES	1.9	14.0	13.3	41.2	29.6	100

Table S3. Diffusion coefficients defived in this study from MD simulations at 298.15 K and 1 bar. Sim stands for simulated values, Exp for literature experimental ones, Error represents the deviation of simulated values from experimental and was obtained according to description in the Sec. 2.1 of the main article. Standard deviations are shown in the parentheses. In some cases the obtained in this work value was compared to a simulated literature one (when no experimental data were found).

Systems	D_{Ch}, 10^{-11} m^2 s^{-1}			D_{Cl}, 10^{-11} m^2 s^{-1}			D_{HBD}, 10^{-11} m^2 s^{-1}			D_{surf}, 10^{-11} m^2 s^{-1}	D_{mic}, 10^{-11} m^2 s^{-1}
	Sim	Exp	Error, %	Sim	Exp/Sim	Error, %	Sim	Exp	Error, %	Sim	Sim
Eth-SDS-m60	0.86(0.02)	2.6[a,b]	67	1.44(0.09)	3.2[a]	55	1.99(0.09)	4.5[a,b]	56	0.39(0.02)	0.11(0.03)
Gly-SDS-m60	0.06(0.01)	0.38[b]	84	0.05(0.01)	0.24[c]	79	0.09(0.01)	0.52[b]	83	0.06(0.01)	0.01(0.01)
Gly-CTAB-m60	0.07(0.02)		82	0.12(0.01)		50	0.10(0.01)		81	0.07(0.04)	0.01(0.01)
Gly-SB3-12-m60	0.06(0.02)		84	0.09(0.01)		62	0.08(0.01)		84	0.02(0.01)	0.003(0.010)
Rel-SDS-m60	0.13(0.03)	0.35[b]	63	0.18(0.01)			0.34(0.02)	0.66[b]	48	0.27(0.05)	0.08(0.02)
Eth-SDS-m120	0.83(0.09)	2.6[a,b]	68	1.3(0.1)	3.2[a]	59	2.08(0.01)	4.5 [a,b]	54	0.16(0.02)	0.03(0.02)
Gly-SDS-m120	0.05(0.01)	0.38[b]	87	0.05(0.01)	0.24[c]	79	0.06(0.01)	0.52 [b]	88	0.04(0.01)	0.002(0.001)
Gly-CTAB-m120	0.05(0.02)		87	0.07(0.01)		71	0.08(0.01)		85	0.04(0.02)	0.01(0.01)
Gly-SB3-12-m120	0.04(0.03)		89	0.05(0.01)		79	0.06(0.01)		88	0.01(0.01)	0.002(0.010)
Rel-SDS-m120	0.10(0.03)	0.35[b]	71	0.13(0.02)			0.21(0.01)	0.66[b]	68	0.11(0.02)	0.01(0.01)

a [1], b [4], c [2]

References

1. Ferreira, E.S.C.; Voroshylova, I.V.; Pereira, C.M.; D. S. Cordeiro, M.N. Improved Force Field Model for the Deep Eutectic Solvent Ethaline: Reliable Physicochemical Properties. *J. Phys. Chem. B* **2016**, *120*, 10124–10137, doi:10.1021/acs.jpcb.6b07233.
2. Ferreira, E.S.C.; Voroshylova, I.V.; Figueiredo, N.M.; Cordeiro, M.N.D.S. Molecular Dynamic Study of Alcohol-Based Deep Eutectic Solvents. *J. Chem. Phys.* **2021**, *155*, 064506, doi:10.1063/5.0058561.
3. Ferreira, E.S.C.; Voroshylova, I.V.; Cordeiro, M.N.D.S. Probing the Interface of Choline Chloride-Based Deep Eutectic Solvent Ethaline with Gold Surfaces: A Molecular Dynamics Simulation Study. *Surf. Interfaces* **2024**, *46*, 104051, doi:10.1016/j.surfin.2024.104051.
4. D'Agostino, C.; Harris, R.C.; Abbott, A.P.; Gladden, L.F.; Mantle, M.D. Molecular Motion and Ion Diffusion in Choline Chloride Based Deep Eutectic Solvents Studied by 1H Pulsed Field Gradient NMR Spectroscopy. *Phys. Chem. Chem. Phys.* **2011**, *13*, 21383–21391, doi:10.1039/C1CP22554E.
